# Oncogenic Role of SRPK2 in Different Types of Cancer: A Systematic Review

**DOI:** 10.1111/jcmm.71177

**Published:** 2026-05-24

**Authors:** Samuel Inácio da Silva Paiva, Bárbara Braga Ferreira, Alexandre Martins Oliveira Portes, Sebastião Felipe Ferreira Costa, Luiz Otávio Guimarães Ervilha, Raoni Pais Siqueira, Juliana Regina Ribeiro de Souza, Luciana Ângelo de Souza, Gustavo Costa Bressan

**Affiliations:** ^1^ Departamento de Bioquímica e Biologia Molecular Universidade Federal de Viçosa Viçosa Minas Gerais Brazil; ^2^ Departamento de Educação Física Universidade Federal de Viçosa Viçosa Minas Gerais Brazil; ^3^ Departamento de Biologia Geral Universidade Federal de Viçosa Viçosa Minas Gerais Brazil; ^4^ Instituto Nacional de Investigação em Mucosas e Pele (INCT Mucosa e Pele) Universidade Federal de Minas Gerais Belo Horizonte Minas Gerais Brazil

**Keywords:** cancer, metastasis, serine/arginine protein kinase 2 (SRPK2), targeted therapy, tumour

## Abstract

A systematic review was conducted to evaluate the available evidence regarding the tumorigenic and metastatic roles of serine/arginine protein kinase 2 (SRPK2) across different cancer types. A range of preclinical studies was included, generally addressing four main aspects: cancer‐related signalling pathways involving SRPK2; its prognostic associations; its impact on metastatic and/or tumour phenotypes; and the antitumor and/or antimetastatic effects resulting from its inhibition. Here, we summarise and discuss the mechanisms through which SRPK2 exerts its oncogenic functions, as well as the therapeutic potential of targeting this kinase. SRPK2 may promote cancer development through its canonical role in alternative splicing, as well as through its involvement in diverse cellular signalling pathways. Moreover, elevated SRPK2 expression across multiple human malignancies consistently correlates with poor clinical outcomes. Collectively, these findings highlight SRPK2 as a promising therapeutic target and potential tumour biomarker.

## 1 Introduction

Alternative splicing (AS) is a finely regulated post‐transcriptional process in which introns are removed from primary messenger RNA (mRNA) transcripts and exons are joined in different combinations, resulting in the generation of distinct mature mRNA isoforms [1]. Under physiological conditions, AS contributes to the regulation of cellular functions and the maintenance of tissue homeostasis [2, 3]. However, when dysregulated, AS can lead to the development of various diseases, including cancer [4, 5].

In cancer, AS dysregulation promotes tumorigenesis by generating oncogenic mRNA isoforms that encode pro‐tumorigenic proteins [6, 7, 8]. These proteins support uncontrolled cell proliferation, evasion of apoptosis, migration, invasion and metabolic reprogramming [9, 10, 11]. In addition, alterations in AS may contribute to resistance to anticancer therapies [11]. Therefore, understanding the molecules involved in AS dysregulation, their roles in cellular signalling pathways and their impact on tumorigenesis and metastasis is essential for deciphering this complex process in cancer biology.

In this context, serine/arginine protein kinase 1 and 2 (SRPK1 and SRPK2) play a central role in AS regulation [12]. These kinases are classically activated by the EGF/PI3K/Akt signalling pathway and function by phosphorylating serine/arginine‐rich (SR) proteins, which regulate splice site selection and facilitate spliceosome assembly [13, 14]. Both SRPK1 and SRPK2 are overexpressed in multiple cancer types and are directly associated with aberrant AS events that drive disease progression [15, 16, 17].

The oncogenic functions of SRPK1 are relatively well characterised. Its overexpression promotes carcinogenesis by enhancing cell proliferation, resistance to apoptosis and chemoresistance [18, 19, 20]. The multifaceted roles of SRPK1 have been comprehensively reviewed elsewhere [21, 22], including evidence that multiple signalling pathways regulate SRPK1 activity and contribute to cancer progression, supporting its relevance as a potential therapeutic target and prognostic biomarker [23].

In contrast, SRPK2 remains less well understood. Although elevated SRPK2 expression has been associated with aggressive tumour phenotypes [17, 24, 25, 26] and involvement in diverse signalling pathways that promote tumour progression [27, 28], a systematic understanding of its oncogenic functions and the effects of its inhibition is still lacking. This systematic review aims to synthesise the available evidence on SRPK2‐related signalling pathways in cancer, their prognostic implications, roles in tumour progression and the potential antitumor and antimetastatic effects resulting from its inhibition.

## 2 Materials and Methods

### 2.1 Protocol and Registration

This systematic review was conducted in accordance with the PRISMA (Preferred Reporting Items for Systematic Reviews) guidelines [29]. The methodological protocol was registered on the Open Science Framework (OSF) platform and is available at: https://doi.org/10.17605/OSF.IO/W34RB.

### 2.2 Guiding Questions

The primary guiding question of this review was: *What is the oncogenic role of SRPK2 in different types of cancer?*

The secondary questions were: (i) SRPK2 promotes what tumorigenic and metastatic impacts; (ii) through which cellular signalling pathways does this kinase act to favour tumour progression and (iii) what antitumor and antimetastatic effects result from its inhibition.

### 2.3 Search Strategy

All studies published up to July 2025 that investigated the oncogenic role of SRPK2 were included. Standardised descriptors from the MeSH and Emtree vocabularies were used to develop the search strategy. The search term, which combined the two descriptors, employed in the database searches was: ((‘Serine‐Arginine Protein Kinase 2’ OR ‘Serine/Arginine Protein Kinase 2’ OR ‘Serine Arginine Protein Kinase 2’ OR SRPK2 OR ‘SRPK 2’) AND (cancer OR adenocarcinoma OR neoplasia OR neoplasm)).

### 2.4 Inclusion and Exclusion Criteria

Inclusion and exclusion criteria were defined using the PICO framework (Table S1). Studies investigating the tumorigenic and metastatic roles of SRPK2 in cancer, the cellular signalling pathways through which SRPK2 promotes tumour progression, or the effects of genetic or pharmacological interventions targeting SRPK2 were included.

Conference papers, review articles and studies addressing SRPK2 in benign or non‐cancer‐related pathological contexts were excluded. Studies focused exclusively on the structural, chemical, or physicochemical characterization of SRPK2 inhibitors without biological validation in cell lines and/or animal models were also excluded. There were no restrictions on the language of the publications.

### 2.5 Study Selection

Studies were retrieved from the Embase, Scopus, ScienceDirect, PubMed and Web of Science databases (Table S2). After duplicate removal, records were subjected to a two‐step screening process. Screening and selection were conducted independently by two authors (Samuel Inácio da Silva Paiva and Bárbara Braga Ferreira). Initially, studies were screened based on title and abstract, followed by full‐text evaluation. Discrepancies were resolved by consensus or, when necessary, by consultation with a third author (Raoni Pais Siqueira). All procedures were performed using Rayyan [30].

### 2.6 Data Extraction

Data extraction from studies was independently performed by two authors (Samuel Inácio da Silva Paiva and Bárbara Braga Ferreira). Extracted data included general study characteristics, SRPK2‐related oncogenic signalling pathways, tumorigenic and metastatic outcomes associated with SRPK2, antitumor and antimetastatic effects resulting from its inhibition and prognostic implications. Data on oncogenic and prognostic outcomes were grouped according to cancer type.

### 2.7 Risk of Bias and Methodological Quality Assessment In Vivo Studies

Risk of bias in in vivo studies was independently assessed by two authors (Alexandre Martins Oliveira Portes and Sebastião Felipe Ferreira Costa), with each bias domain evaluated according to SYRCLE's (Systematic Review Centre for Laboratory Animal Experimentation) tool [31]. Review Manager software was used for visualisation.

Methodological quality was assessed using the CAMARADES (Collaborative Approach to Meta‐Analysis and Review of Animal Data from Experimental Studies) checklist [32], which comprises 10 items (maximum score: 10 points). Studies were classified as low (1–3 points), moderate (4–7 points), or high quality (8–10 points). Analyses were conducted independently by the same two authors (Alexandre Martins Oliveira Portes and Sebastião Felipe Ferreira Costa).

### 2.8 Disclosure of Artificial Intelligence Use

Grammarly, Gemini (Google) and ChatGPT (OpenAI) were used to improve the clarity and grammar of the text. All AI‐generated suggestions were critically reviewed and incorporated, when appropriate, into the final text by the authors, who retain full responsibility for the content of this manuscript.

## 3 Results and Discussion

### 3.1 Search Results and Study Characteristics

**FIGURE 1:**
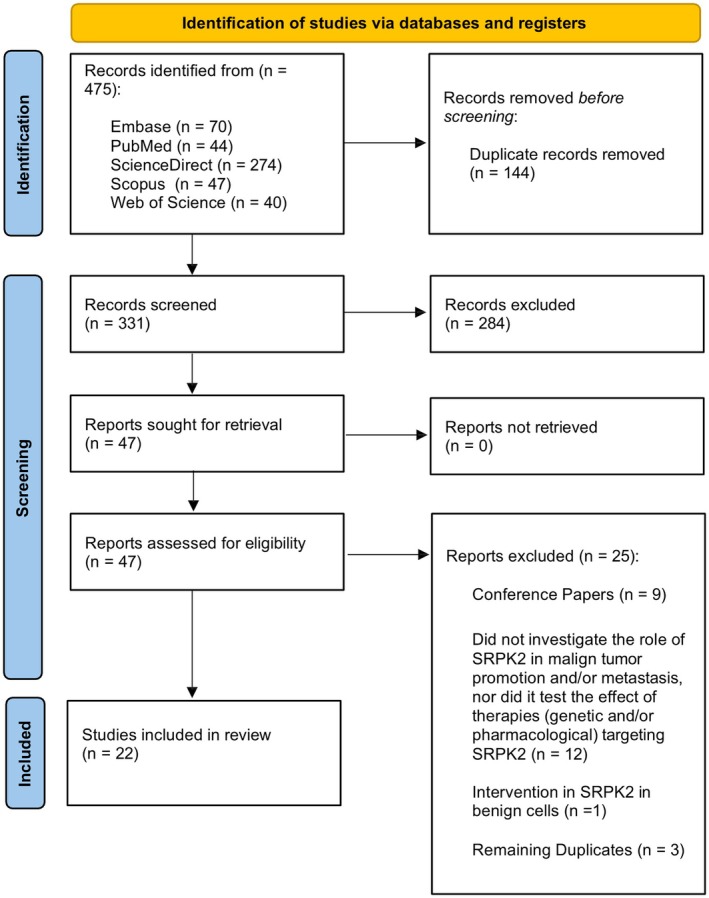
PRISMA flow diagram for literature search and study selection.

The initial database search (Embase, PubMed, ScienceDirect, Scopus and Web of Science), covering publications up to July 2025, identified 475 records. After duplicate removal, 331 studies entered in Screening Phase 1 (title and abstract reading), resulting in the exclusion of 284 records. The remaining 47 articles underwent full‐text reading (Phase 2), leading to the exclusion of 25 studies. Ultimately, 22 studies met the eligibility criteria and were included in this systematic review. The complete process, including reasons for exclusion, is presented in the PRISMA flow diagram (Figure 1).

Regarding study characteristics, 18 studies used human cancer cell lines, including breast, bladder, colon, head and neck, leukaemia, liver, pancreatic, prostate and lung cancers [17, 25, 26, 27, 28, 33, 34, 35, 36, 37, 38, 39, 40, 41, 42, 43, 44, 45]. Three studies used murine melanoma cell lines [15, 46, 47] (Table 1), while one study did not employ cell lines [24] (Table 1). Eight studies used animal models [15, 17, 25, 33, 34, 38, 46, 47].

**TABLE 1:** Summary of carcinogenic signalling pathways related to SRPK2 activity.

Author, year	Cancer type	Signalling pathway	Tumour/metastatic outcomes	Antitumor/antimetastatic outcomes	Cell type	Animal model	SRPK2 intervention method
McClellan et al. 2022 [35]	Breast cancer	IGF‐1R/mTORC1/SRPK2/SRSF1/FASN	In vitro: ↑FASN mRNA expression and stability, ↑palmitate synthesis	In vitro: ↓FASN mRNA expression and stability, ↓palmitate synthesis	Human breast cancer lineage	N/A	Knockdown, pharmacological inhibition (SRPIN340)
Liu et al. 2021 [17]	Colon cancer	SRPK1/2/SRSF1/MKNK2b	In vitro: ↑MKNK2b, ↑proliferation, ↑colony formation. In vivo: ↑tumour growth, ↑Ki67	In vitro: ↓MKNK2b, ↓proliferation, ↓colony formation. In vivo: ↓tumour growth, ↓Ki67	Human colon cancer lineage	Balb/c nude mice	Knockdown, kinase‐dead mutants, pharmacological inhibition (SRPIN340), overexpression
Wang et al. 2020 [36]	Colon cancer	SRPK2/Numb/p53	In vitro: ↑chemoresistance, ↑migration, ↑invasion	In vitro: ↓chemoresistance, ↓ migration, ↓ invasion	Human Colon Cancer Lineage	N/A	Knockdown, overexpression
Wang et al. 2016 [33]	Colon cancer	SRPK2/B‐RAF/ERK	In vitro: ↑cell growth, ↑cell migration. In vivo: ↑tumour growth	N/A	Human colon cancer lineage	Nude mice	Knockdown, overexpression
Yang et al. 2018 [37]	Colon Cancer	SRPK2/TWIST	N/A	In Vitro: ↓TWIST, ↓vimentin, ↑E‐cadherin	Human colon cancer lineage	N/A	Knockdown
Lee et al. 2017 [38]	Diverses: bladder, breast, lung	mTORC1/S6K1/SRPK2	N/A	In vitro: ↓expression of lipogenic genes, ↓cell growth. In vivo: ↓tumour growth	Human bladder, breast and lung cancer lineage	Nude CD‐1 mice, CB17‐scid mice	Knockdown, pharmacological inhibition (SRPIN340), insulin stimulation
Radhakrishnan et al. 2016 [39]	Head and neck cancer	N/A	N/A	In vitro: ↓colony formation, ↓invasion	Human head and neck cancer lineage	N/A	Knockdown, pharmacological inhibition (SRPIN340)
Jang et al. 2008 [43]	Leukaemia	SRPK2/acinus/cyclin A1	In vitro: ↑cyclin A1, accumulation in the G2‐M phase, ↑proliferation	In vitro: ↓cyclin A1, cell cycle arrest in the G1 phase, ↓proliferation	Human leukaemia lineage	N/A	Knockdown, overexpression, recombinant protein stimulation (EGF)
Siqueira et al. 2015 [41]	Leukaemia	N/A	N/A	In vitro: ↓cell viability, ↑apoptosis, ↓MAP2K1, ↓VEGF, ↑FAS	Human leukaemia lineage	N/A	Pharmacological inhibition (SRPIN340)
Siqueira et al. 2017 [42]	Leukaemia	N/A	N/A	In vitro: ↓cell viability, ↑apoptosis, ↑autophagy, ↓MAP2K1, ↓MAP2K2, ↓VEGF, ↓RON	Human leukaemia lineage	N/A	Pharmacological inhibition (SRPIN340, SRVICs)
Siqueira et al. 2020 [40]	Leukaemia	AKT/SRPK2	N/A	In vitro: Synergism in SRPK and AKT inhibition (T‐ALL), ↓cell viability, ↑apoptosis	Human leukaemia lineage	N/A	Pharmacological inhibition (SRPIN340)
Zhou et al. 2022 [28]	Leukaemia	BP1/SGMS1‐AS1/miR‐181d‐5p/SRPK2	In vitro: ↑ SRPK2	N/A	Human leukaemia lineage	N/A	Indirect modulation via overexpression of the BP1 protein
Lu et al. 2015 [44]	Liver cancer	Hsp90/SRPK2/Numb	In vitro: ↑Numb PRR^L^	In vitro: ↓Numb PRR^L^ ↑Numb PRR^S^	Human liver cancer lineage	N/A	Knockdown
Wu et al. 2024 [26]	Liver cancer	LINC01446/SRPK2/SRSF1/VEGFA_165_	In vitro: ↑cell growth, ↑colony formation, ↑VEGFA_165_, ↑sorafenib resistance	In vitro: ↓cell growth, ↓colony formation, ↓VEGFA_165_, ↓sorafenib resistance	Human liver cancer lineage	N/A	Knockdown, pharmacological inhibition (SRPKIN‐1), overexpression
Gout et al. 2012 [24]	Lung cancer	N/A	Ex vivo: ↑SRPK2 in non‐small cell lung cancer (NSCLC) ↑pSRSF2 (NSCLC, ADC subtype)	N/A	N/A	N/A	N/A
Li et al. 2019 [25]	Lung Cancer	SRPK2/SC35/E2F1	In vitro: cell cycle progression, ↑proliferation, ↑cyclin E1, ↑p45^SKP2^	In vitro: cell cycle arrest, ↓proliferation. In vivo: ↓tumour growth, ↓Ki67	Human lung cancer lineage	Balb/c nude mice	Knockdown, overexpression
Caetano et al. 2022 [15]	Melanoma	N/A	N/A	In vitro: ↓proliferation, ↓invasion, ↓actin polymerisation. In vivo: ↓tumour growth, ↓lung metastasis	Murine melanoma lineage	C57BL/6	Knockout
Moreira et al. 2018 [47]	Melanoma	N/A	N/A	In vitro: ↓migration, ↓invasion, ↓adhesion, ↓colony formation. In vivo: ↓lung metastasis	Murine melanoma lineage	C57BL/6	Pharmacological inhibition (SRPIN340, SRVICs)
Moreira et al. 2022 [46]	Melanoma	N/A	N/A	In vivo: ↓tumour growth, ↓metastasis, ↑apoptosis, ↓N‐cadherin, ↑E‐cadherin. ↑CD4 and CD8 infiltration, ↑expression of pro‐inflammatory cytokines, stimulation of CD80 and CD86. In vitro: ↑MHCI, ↑MHCII, attraction of splenocytes	Murine melanoma lineage	C57BL/6	Pharmacological inhibition (SRPIN340)
Fonteneau et al. 2022 [27]	Pancreatic cancer	IGF‐1/IGF1‐R/S6K1/SRPK2	In vitro: ↑SG formation	In vitro: ↓SG formation	Human pancreatic cancer lineage	N/A	Knockdown, phosphodeficient mutants, phosphomimic mutants, stimulation with recombinant protein (IGF‐1)
Wang et al. 2019 [45]	Pancreatic cancer	SRPK2/Numb/p53	In vitro: ↑chemoresistance, ↑migration, ↑invasion	In vitro: ↓chemoresistance, ↓migration, ↓invasion	Human pancreatic lineage	N/A	Knockdown, overexpression
Zhuo et al. 2018 [34]	Prostate cancer	N/A	In vitro: cell cycle progression, ↑proliferation, ↑migration, ↑invasion, ↓apoptosis. In vivo: ↑tumour growth	N/A	Human prostate cancer lineage	Nude mice	Overexpression

Abbreviations: ↓, decrease; ↑, increase; N/A, not applicable.

Eight studies induced SRPK2 overexpression, and one study employed a phosphomimetic mutant [17, 25, 26, 33, 34, 36, 43, 45] (Table 1). Two studies used kinase‐dead or phosphodeficient mutants [17, 27] (Table 1). One study indirectly stimulated SRPK2 expression [28] (Table 1), while three studies evaluated SRPK2 activity using recombinant protein [27, 38, 43] (Table 1).

Finally, 14 studies performed genetic interventions targeting SRPK2 expression, including knockdown or knockout approaches [15, 17, 25, 26, 27, 33, 35, 36, 37, 38, 39, 43, 44, 45] and 10 studies investigated pharmacological inhibitors [17, 26, 35, 38, 39, 40, 41, 42, 46, 47]. Among these, five combined pharmacological and genetic approaches to validate SRPK2 involvement in oncogenic processes, while the remaining five focused on the effects of pharmacological inhibition (Table 1).

### 3.2 Prognostic Implications

Elevated SRPK2 expression has been consistently associated with poor clinical outcomes across multiple solid tumours (Table S3). Higher SRPK2 levels correlate with advanced pathological stage and higher tumour grade in colon, pancreatic, and prostate cancers, as well as in lung adenocarcinoma [24, 34, 36, 45].

SRPK2 is also linked to metastatic progression, including lymph node dissemination in colon and pancreatic cancers, liver metastasis in pancreatic carcinoma [36, 45] and metastasis in prostate cancer [34]. In melanoma, high SRPK2 expression is associated with poor prognosis [15], reduced inflammatory response scores, and enrichment of pathways related to Wnt/β‐catenin signalling and epithelial–mesenchymal transition (EMT) [46].

Collectively, these findings support SRPK2 as a relevant prognostic factor and highlight the need for further evaluation of its potential clinical applicability.

### 3.3 Oncogenic Roles of SRPK2

Table 1 summarises the tumorigenic and metastatic mechanisms associated with SRPK2, the signalling pathways involved, and the antitumor and antimetastatic effects observed following its targeting in vitro and in vivo. These mechanisms are discussed in detail in the following sections (Sections 3.3.1, 3.3.4 and 4).

#### 3.3.1 Regulation of Cell Cycle, Proliferation and Migration Mediated by SRPK2

**FIGURE 2:**
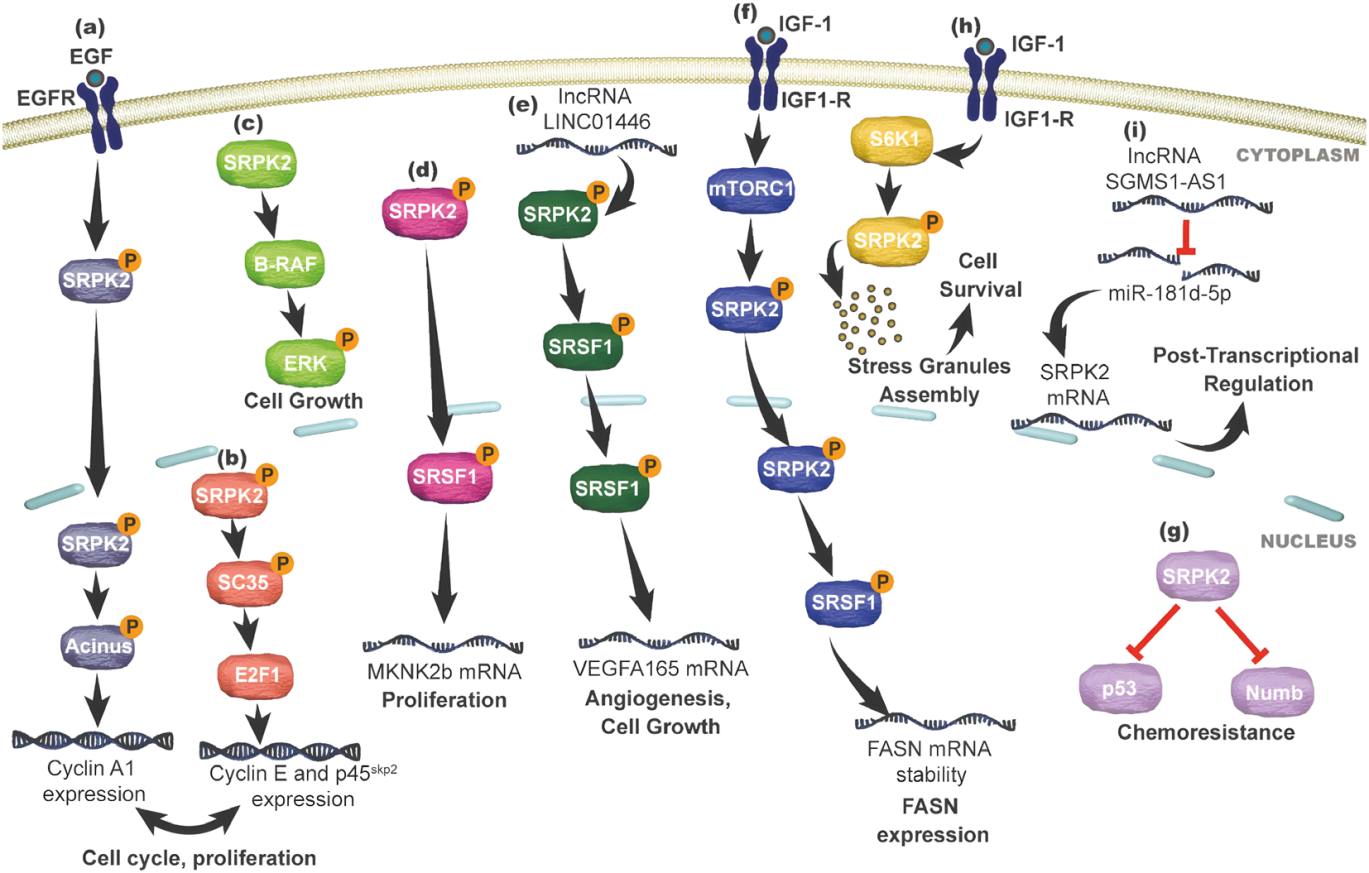
Proposed integrative model of the SRPK2 regulatory network and its dysregulation in cancer cell signalling. The schematic illustrates a conceptual framework highlighting the functional diversity of SRPK2. Under physiological conditions, SRPK2 contributes to molecular homeostasis by regulating key cellular processes. However, its dysregulation activates a complex signalling network across different tumour contexts: (a, b) In leukaemia and lung cancer cells, SRPK2 modulates the expression of genes associated with cell cycle progression and proliferation [25, 43]. (c) In colon cancer, SRPK2 promotes cell growth through modulation of ERK phosphorylation [33]. (d–f) SRPK2 regulates alternative splicing events in breast, colon and liver cancer cells, favouring the production of pro‐tumorigenic mRNA isoforms that enhance angiogenesis, proliferation and cell growth, while also supporting tumour lipid metabolism via FASN expression [17, 26, 35]. (g) The kinase negatively regulates Numb and the tumour suppressor p53 in colon and pancreatic carcinomas, contributing to resistance to chemotherapeutic agents [36, 45]. (h) In pancreatic cancer, SRPK2 associates with stress granules and promotes their assembly, supporting tumour cell survival under stress conditions [27]. (i) In leukaemia, the lncRNA SGMS1‐AS1 modulates the post‐transcriptional regulation of SRPK2 by sequestering miR‐181d‐5p. Inhibition of this microRNA stabilises SRPK2 mRNA, protecting it from translational repression [28].

SRPK2 regulates cell cycle progression through distinct signalling mechanisms, depending on the tumour type. In leukaemia, EGF‐induced activation of SRPK2 promotes Acinus phosphorylation and increases Cyclin A1 expression, resulting in G2/M phase accumulation (Figure 2a) [43]. In lung cancer, SRPK2‐mediated phosphorylation of SC35 activates the transcription factor E2F1, leading to increased expression of Cyclin E and p45^Skp2^ (Figure 2b) [25].

In colon cancer, SRPK2 overexpression promotes cell growth via ERK phosphorylation through its interaction with B‐RAF (Figure 2c) [33], while in prostate carcinoma, it enhances cell cycle progression and proliferation and suppresses apoptosis [34]. In colorectal cancer, elevated SRPK1/2 expression leads to SRSF1 phosphorylation and increased production of the oncogenic isoform MKNK2b, which is associated with enhanced proliferation (Figure 2d) [17]. This effect is reversed by the expression of catalytically inactive mutants, genetic knockdown, or pharmacological inhibition with SRPIN340 [17].

In liver cancer, SRPK2 interacts with the chaperone HSP90 to regulate Numb AS, favouring the long isoform (PRR^L^)—associated with increased invasion and migration—over the short isoform (PRR^S^) [44]. Additionally, in colon cancer, SRPK2 upregulation promotes cell migration, whereas its silencing reduces cell motility [33].

Functional disruption of SRPK2 impairs cell proliferation and cell cycle progression across multiple tumour models. Genetic targeting of SRPK2 induces G1 phase arrest in leukaemia and reduces proliferation and invasion in melanoma [15, 43], consistent with findings from pharmacological inhibition studies using SRPK inhibitors [40, 41, 42, 47]. Furthermore, SRPK2 depletion in colon and lung xenograft models results in reduced tumour volume and decreased Ki‐67 expression [17, 25].

#### 3.3.2 Role of SRPK2 in Tumour Lipid Metabolism

Beyond its role in regulating cell cycle progression, proliferation and migration, SRPK2 also contributes to metabolic reprogramming in cancer cells, particularly by modulating lipid biosynthesis pathways.

SRPK2 has been identified as a regulator of de novo lipid synthesis in breast, bladder and lung cancer cell lines [35, 38]. In triple‐negative breast cancer cells, exposure to IGF‐1 activates the mTORC1 pathway, leading to SRPK2 phosphorylation, activation and nuclear translocation. This process enhances SRSF1 phosphorylation and promotes its interaction with FASN mRNA, resulting in increased transcript stability, elevated FASN expression and enhanced de novo palmitate synthesis (Figure 2f) [35]. Conversely, treatment with the SRPK inhibitor SRPIN340 or genetic knockdown attenuates this activity and reduces FASN expression levels [35].

Consistent with these findings, similar inhibitory effects on lipogenic enzyme expression and cell growth have been observed in bladder cancer cell lines following treatment with SRPIN340 [38]. In vivo, both pharmacological inhibition and xenograft models using SRPK2‐silenced cells resulted in reduced tumour growth in bladder and lung cancer models [38].

#### 3.3.3 Implications of SRPK2 in EMT and Invasion

In addition to its roles in metabolic reprogramming, SRPK2 also contributes to tumour cell plasticity and invasive behaviour. In colon cancer, SRPK2 expression promotes EMT, characterised by increased levels of vimentin and TWIST and reduced E‐cadherin expression, which can be reversed upon genetic silencing of the kinase [37]. Consistently, treatment of melanoma‐bearing mice with the SRPK inhibitor SRPIN340 shifts this profile, decreasing N‐cadherin and increasing E‐cadherin expression [46]. However, as SRPIN340 inhibits both SRPK1 and SRPK2, the specific contribution of SRPK2 to the N‐/E‐cadherin expression pattern in melanoma requires further investigation using selective genetic targeting approaches.

Genetic targeting of SRPK2 using CRISPR/Cas9 or RNA interference produces effects comparable to pharmacological inhibition in invasion assays across colorectal, head and neck, melanoma and pancreatic cancer models [15, 36, 39, 45]. In in vivo melanoma models, both pharmacological inhibition and inoculation of SRPK2 knockout cells reduce pulmonary metastatic burden, consistent with the reduced invasive capacity observed in vitro [15, 46, 47].

#### 3.3.4 Cooperation of SRPK2 in Cell Survival and Therapeutic Resistance

Beyond its roles in tumour cell plasticity and invasion, SRPK2 also contributes to cell survival under stress conditions and to resistance to anticancer therapies. In colon and pancreatic cancer cell lines, SRPK2 overexpression decreases sensitivity to 5‐fluorouracil, cisplatin, gemcitabine and oxaliplatin [36, 45]. This chemoresistant phenotype is mechanistically linked to the downregulation of the tumour suppressors p53 and Numb (Figure 2g), whose expression and function are restored following kinase silencing, thereby resensitizing tumour cells to treatment [36, 45].

Consistently, SRPK2 regulates the formation of stress granules (SGs)—cytoplasmic structures associated with cell survival under stress—in pancreatic carcinoma cells [27]. This process is mediated by the IGF‐1R/S6K1 pathway, which promotes SRPK2 phosphorylation and its accumulation within these protective structures (Figure 2h) [27]. Disruption of this axis, through S6K1 inhibition, SRPK2 knockdown, or the use of phosphodeficient mutants, impairs SG assembly, whereas the expression of wild‐type SRPK2 or phosphomimetic mutants restores SG formation [27].

These findings are correlated by studies investigating SRPK inhibitors. Pharmacological treatment of leukaemia cells increases sensitivity to AKT inhibitors and vincristine, while also enhancing autophagy and apoptosis mediated by the upregulation of FAS and RON, and the downregulation of MAP2K1 and MAP2K2 [41, 42]. Similarly, treatment with SRPIN340 enhances the immunogenicity of melanoma cells by increasing the infiltration of CD4^+^ and CD8^+^ T lymphocytes into tumours and promoting the expression of pro‐inflammatory mediators, including IL‐6, TNF‐α, IL‐1β, IL‐1ra and iNOS [46]. Although pharmacological inhibitors present inherent limitations, and considering the potentially more prominent role of SRPK2 relative to SRPK1 in melanoma and leukaemia [15, 41], these findings provide additional support for the involvement of SRPK2 in tumour cell survival and therapeutic resistance. These hypotheses certainly warrant further validation in future studies.

Collectively, the evidence discussed in Sections 3.3.1, 3.3.4 highlights the multifaceted role of SRPK2 in cancer, encompassing the regulation of cell cycle progression, metabolic reprogramming, EMT, invasion and therapeutic resistance. These diverse oncogenic functions suggest that sustained SRPK2 activity is a critical determinant of tumour progression across multiple cancer types. Given this broad functional impact, understanding the molecular mechanisms that regulate SRPK2 overexpression becomes essential to fully elucidate its role in cancer biology and to identify additional potential points of therapeutic intervention.

## 4 Regulation of SRPK2 Expression and Stability in Cancers

The maintenance of elevated SRPK2 levels has been shown to be regulated by distinct molecular mechanisms, depending on the biochemical tumour context. Regulatory mechanisms involving non‐coding RNAs have been described in leukaemia, where the oncoprotein BP1 increases the expression of the lncRNA SGMS1‐AS1 [28]. This lncRNA sequesters miR‐181d‐5p, thereby preventing the translational repression of SRPK2 mRNA (Figure 2i) and increasing SRPK2 expression [28].

In hepatic carcinoma cells, direct interaction with the lncRNA LINC01446 stabilises SRPK2, increasing its protein half‐life and overall levels [26]. This interaction‐dependent stabilisation promotes SRSF1 phosphorylation and its binding to VEGF‐A pre‐mRNA, resulting in increased VEGF‐A_165_ expression and secretion, as well as enhanced angiogenesis (Figure 2e) [26]. These molecular events are accompanied by increased cell growth, colony formation and resistance to sorafenib [26]. Moreover, silencing of LINC01446 reduces SRPK2 levels, while SRPK2 knockdown disrupts the interaction between SRSF1 and VEGF‐A pre‐mRNA, leading to decreased VEGF‐A_165_ expression, reduced cell growth and colony formation and increased sensitivity to sorafenib [26].

## 5 Concluding Remarks

SRPK2 drives oncogenesis and metastasis through multiple signalling pathways, both dependent on and independent of its role in pre‐mRNA splicing. It promotes EMT [37], enhances tumour cell invasiveness, stimulates cell migration and proliferation and contributes to chemoresistance by supporting cell survival under stress conditions and suppressing apoptosis [27, 33, 34, 36, 45]. Taken together, these findings position SRPK2 as a central integrator of key oncogenic processes, linking AS regulation to broader signalling networks that sustain tumour progression.

The involvement of SRPK2 in tumorigenesis and metastatic mechanisms across diverse signalling pathways underscores its relevance as a therapeutic target. This is supported by studies demonstrating that genetic targeting of SRPK2, through knockdown or knockout approaches, suppresses tumour growth and metastatic phenotypes [15, 17, 25, 38]. Moreover, although highly selective SRPK2 inhibitors are not yet available, pharmacological inhibition of SRPK recapitulates many of the effects observed with genetic approaches. These interventions have shown consistent efficacy in preclinical in vitro and in vivo models, including reduced tumour growth [38, 46], decreased cell proliferation [41, 42], increased tumour infiltration by CD4^+^ and CD8^+^ T lymphocytes [46, 47] and enhanced sensitivity to conventional chemotherapeutic agents [17, 26, 35, 40, 41, 42].

In addition to its therapeutic potential, SRPK2 has emerged as a promising prognostic biomarker across multiple malignancies [15, 17, 24, 25, 34, 36, 45, 46]. Its consistent association with adverse clinical outcomes, such as metastasis, disease recurrence and reduced overall survival, highlights its potential clinical relevance and supports further investigation in translational settings.

Overall, the evidence presented in this systematic review supports SRPK2 as a multifunctional oncogenic regulator with significant potential as both a therapeutic target and a prognostic biomarker. Future efforts should focus on the development of highly selective SRPK2 inhibitors and on the validation of its potential prognostic utility in large‐scale studies, which will be essential for translating these findings into improved patient outcomes.

## 6 Assessments of Risk of Bias and Methodological Quality of In Vivo Studies

**FIGURE 3:**
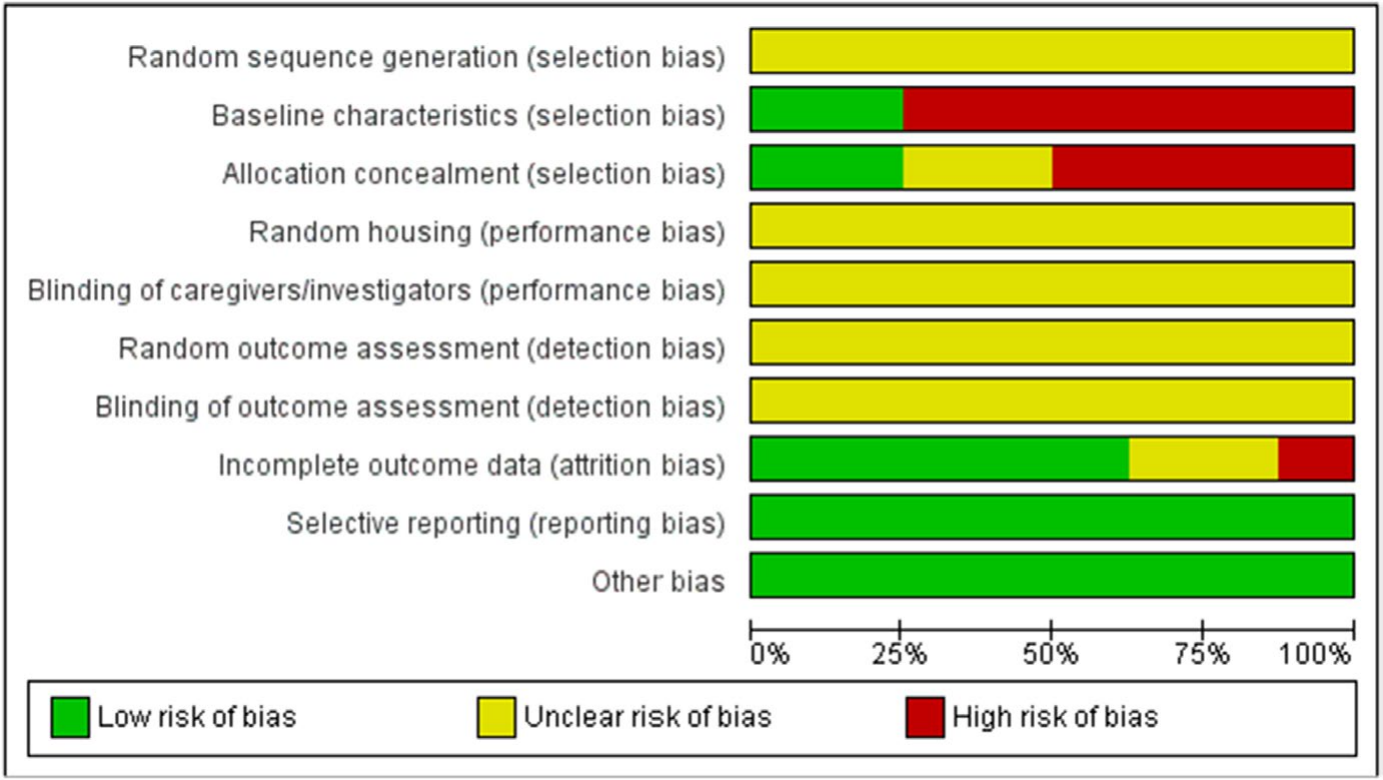
Risk of bias assessment of the included in vivo studies. Summary chart illustrating the methodological quality and reporting of risk of bias domains for the studies included in the systematic review, based on the SYRCLE tool.

For these assessments, only studies that modulated SRPK2 in animal models through genetic interventions [15, 17, 25, 33, 34, 38] or using SRPK inhibitors [38, 46, 47] were considered. Results of the risk of bias assessment are presented in Figures 3 and S1. The risk of selection bias was rated as unclear in all studies due to the absence of reported random sequence generation. Most studies (*n* = 6) were classified as having a high risk of bias regarding baseline characteristics (e.g., body mass, age and sex), owing to incomplete reporting. Exceptions were two studies classified as low risk, in which animals served as their own controls [33, 34].

Allocation bias was similarly rated as unclear or high risk in most studies, with only two studies [33, 34] classified as low risk for the same reason (self‐controlled experimental design). All studies were rated as unclear for performance bias, due to the lack of reporting on random housing and blinding of caregivers and investigators and for detection bias, due to the absence of information on random outcome assessment or blinding of evaluators. Regarding attrition bias, the majority of studies (*n* = 5) were classified as low risk, as they reported the number of animals in both the Methods and Results sections [17, 25, 33, 34, 46]. In addition, all studies were rated as low risk for selective reporting and other potential sources of bias.

Overall, the risk of bias assessment indicates that studies investigating SRPK2 modulation in animal models present important reporting limitations, particularly regarding randomisation procedures and blinding. These limitations, together with insufficient control of baseline characteristics in most studies (with the exception of self‐controlled designs), result in predominantly unclear or high risk of selection, performance and detection biases. In contrast, attrition and selective reporting biases appear to be consistently low across the analysed studies. Regarding methodological quality, all studies were classified as having moderate quality according to the CAMARADES criteria, with scores ranging from 5 to 6 points (Table S4).

In summary, future studies using animal models should rigorously report randomisation and blinding procedures to improve the reliability and reproducibility of the evidence.

## 7 Limitations

This systematic review has limitations that should be considered when interpreting and extrapolating the findings.

First, most of the included studies investigated tumorigenic and metastatic phenotypes in vitro and should therefore be interpreted with caution. It is well established that tumour cells cultured in vitro do not fully recapitulate the complexity of the tumour microenvironment or adequately represent tumour heterogeneity [48, 49]. In addition, there is currently no validated tool for assessing the risk of bias in in vitro studies included in systematic reviews [50]. Consequently, the absence of a comprehensive methodological evaluation, including quality and risk‐of‐bias assessments, limits the generalisability of these findings.

Studies employing animal models were also included. Among these, syngeneic models [15, 46, 47], in which tumour cells from the same species are inoculated [51], warrant particular consideration. Although these models are valuable for investigating immune responses, since they do not require immunosuppression, they do not adequately reflect the heterogeneity of human tumours [51]. Xenograft models [17, 25, 33, 34, 38], which involve the use of tumour cells from different species (typically human cells), generally require immunosuppression and, despite more closely approximating certain aspects of human tumour heterogeneity [51, 52], should also be interpreted with caution. These models may fail to capture the complex interactions between tumours and the immune system and often present a tumour microenvironment that differs from that observed in patients with intact immune function [53, 54]. Furthermore, considering the risk of bias and methodological quality assessments presented (Figure 3, Figure S1 and Table S4), more detailed reporting of experimental procedures in animal studies is strongly encouraged to reduce potential sources of bias.

Finally, the pharmacological inhibitors SRPIN340, SRVICs and SRPKIN‐1 target both SRPK1 and SRPK2 [41, 42, 47, 55]. Therefore, the effects observed with these compounds cannot be exclusively attributed to SRPK2 inhibition, representing an important limitation in the interpretation of pharmacological evidence.

## Author Contributions

**Bárbara Braga Ferreira:** formal analysis, writing – review and editing, investigation. **Luiz Otávio Guimarães Ervilha:** writing – review and editing, methodology. **Sebastião Felipe Ferreira Costa:** formal analysis, writing – review and editing. **Alexandre Martins Oliveira Portes:** formal analysis, writing – review and editing. **Juliana Regina Ribeiro de Souza:** writing – review and editing. **Samuel Inácio da Silva Paiva:** conceptualization, methodology, formal analysis, writing – original draft, investigation, writing – review and editing. **Luciana Ângelo de Souza:** writing – review and editing. **Raoni Pais Siqueira:** supervision, writing – review and editing. **Gustavo Costa Bressan:** supervision, writing – review and editing, resources, validation, funding acquisition.

## Funding

This work was supported by Coordenação de Aperfeiçoamento de Pessoal de Nível Superior (CAPES) (grant: finance code—001), Fundação de Amparo à Pesquisa do Estado do Minas Gerais (FAPEMIG) (grants: APQ‐01084‐21; RED00096‐22 [Rede de Investigação em Mucosas e Pele]; BPD‐00827‐22; RED‐00067‐23 [Rede de Imunobiológicos] and APQ‐05143‐23) and Conselho Nacional de Desenvolvimento Científico e Tecnológico (CNPq) (grants: 408484/2024‐1 [Instituto Nacional de Investigação em Mucosas e Pele, INCT Mucosa e Pele] and 311163/2022‐0).

## Conflicts of Interest

The authors declare no conflicts of interest.

## Supporting information


**Figure S1:** Risk of bias assessment of the included studies according to the SYRCLE tool. The symbol (+) indicates low risk of bias, (−) indicates no high risk of bias and (?) indicates unclear risk of bias.
**Table S1:** PICO statement.
**Table S2:** Search strategy.
**Table S3:** Prognostic implications.
**Table S4:** CAMARADES assessment of the methodological quality of the included studies.

## Data Availability

The data that support the findings of this study are available from the corresponding author upon reasonable request.
