# Supplementary material for: Oncogenic Role of SRPK2 in Different Types of Cancer: A Systematic Review
Source: J Cell Mol Med. 2026 May 24;30(10):e71177. doi: 10.1111/jcmm.71177 (PMC13239542; doi:10.1111/jcmm.71177)
Supplement: Supplementary file 1 — Figure S1: Risk of bias assessment of the included studies according to the SYRCLE tool. The symbol (+) indicates low risk of bias, (−) indicates no high risk of bias and (?) indicates unclear risk of bias. Table S1: PICO statement. Table S2: Search strategy. Table S3: Prognostic implications. Table S4: CAMARADES assessment of the methodological quality of the included studies. [file JCMM-30-e71177-s001.docx]

**Supporting Information**

**Supplemental Table 1 (Table S1).** PICO statement

| PICO table | |  |
| --- | --- | --- |
| Population (P) | Malignant tumor cells from different lines. Animal models of cancer. Tumor tissue samples from patients. | |
| Intervention (I) | Targeted modulation of SRPK2 expression or activity by recombinant protein, overexpression, knockdown, knockout, pharmacological inhibition, or analysis of its expression levels. | |
| Comparison (C) | Control cells or wild-type cells. Control animals. Non-tumor tissue samples. | |
| Outcome (O) | Tumor and metastatic phenotypic impacts of SRPK2. Cancer cell signaling pathways involving SRPK2. Antitumor and/or antimetastatic results. |  |

**Supplemental Table 2 (Table S2).** Search Strategy

| Database | Search term | Articles found | Date of search | Time |
| --- | --- | --- | --- | --- |
| Embase | (("Serine-Arginine Protein Kinase 2” OR “Serine/Arginine Protein Kinase 2” OR “Serine Arginine Protein Kinase 2” OR SRPK2 OR “SRPK 2”) AND (cancer OR adenocarcinoma OR neoplasia OR neoplasm)) | (*n* = 70) | 07/14/2025 | 09h30 |
| PubMed | (("Serine-Arginine Protein Kinase 2” OR “Serine/Arginine Protein Kinase 2” OR “Serine Arginine Protein Kinase 2” OR SRPK2 OR “SRPK 2”) AND (cancer OR adenocarcinoma OR neoplasia OR neoplasm)) | (*n* = 44) | 07/14/2025 | 09h28 |
| ScienceDirect | (("Serine-Arginine Protein Kinase 2” OR “Serine/Arginine Protein Kinase 2” OR “Serine Arginine Protein Kinase 2” OR SRPK2 OR “SRPK 2”) AND (cancer OR adenocarcinoma OR neoplasia OR neoplasm)) | (*n* = 274) | 07/14/2025 | 09h36 |
| Scopus | (("Serine-Arginine Protein Kinase 2” OR “Serine/Arginine Protein Kinase 2” OR “Serine Arginine Protein Kinase 2” OR SRPK2 OR “SRPK 2”) AND (cancer OR adenocarcinoma OR neoplasia OR neoplasm)) | (*n* = 47) | 07/14/2025 | 09h31 |
| Web of Science | (("Serine-Arginine Protein Kinase 2” OR “Serine/Arginine Protein Kinase 2” OR “Serine Arginine Protein Kinase 2” OR SRPK2 OR “SRPK 2”) AND (cancer OR adenocarcinoma OR neoplasia OR neoplasm)) | (*n* = 40) | 07/14/2025 | 09h32 |

**Supplemental Table 3 (Table S3).** Prognostic Implications

| Author, Year | Cancer Type | Prognostic implications |
| --- | --- | --- |
| Liu et al., 2021 [[17](https://doi.org/10.1186/s13046-021-01877-y)] | Colon cancer | ↑SRPK1/2 in tumor tissues ↓overall survival |
| Wang et al., 2020 [[36](https://doi.org/10.1042/bsr20191488)] | Colon cancer | ↑SRPK2 in tumor tissues, ↑tumor differentiation, ↑T stage, lymph node metastasis, and advanced UICC stage |
| Gout et al., 2012 [[24](https://doi.org/10.1371/journal.pone.0046539)] | Lung Cancer | ↑SRPK2 in tumor tissues, most advanced stage of lung adenocarcinoma |
| Li et al.,  2019 [[25](https://pmc.ncbi.nlm.nih.gov/articles/PMC6945924/)] | Lung Cancer | ↑SRPK2 in tumor tissues, ↓overall survival |
| Caetano et al. 2022 [[15](https://doi.org/10.3389/fgene.2022.979735)] | Melanoma | ↑SRPK1/2 in single-cell analysis, indicator of poor prognosis |
| Moreira et al., 2022 [[45](https://doi.org/10.1016/j.bcp.2022.115161)] | Melanoma | ↑SRPK2 in single-cell analysis, ↓ inflammatory response score, enrichment of Wnt/β-catenin and epithelial/mesenchymal transition |
| Wang et al., 2019 [[47](https://doi.org/10.1111/febs.14778)] | Pancreatic cancer | ↑SRPK2 in tumor tissues, ↑T stage, ↓overall survival, lymph node and liver metastasis, advanced UICC stage |
| Zhuo et al., 2018 [[30](https://doi.org/10.1016/j.biopha.2018.03.079)] | Prostate cancer | ↑SRPK2 in tumor tissues, ↑tumor grade, advanced pathological stage, tumor metastasis, ↓biochemical recurrence-free survival |

↑ increase; ↓ decrease

**Supplemental Figure 1 (Figure S1).**


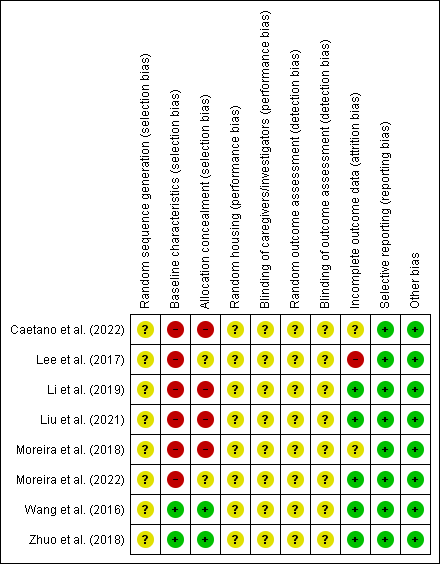


**Figure S1.** **Risk of bias assessment of the included studies according to the SYRCLE tool**. The symbol **(+)** indicates low risk of bias, **(-)** indicates no high risk of bias, and **(?)** indicates unclear risk of bias.

**Supplemental Table 4 (Table S4).** CAMARADES assessment of the methodological quality of the included studies

|  | Study | (1) | (2) | (3) | (4) | (5) | (6) | (7) | (8) | (9) | (10) | Score |
| --- | --- | --- | --- | --- | --- | --- | --- | --- | --- | --- | --- | --- |
|  | Caetano et al. (2022) [[15](https://doi.org/10.3389/fgene.2022.979735)] | ✔ | ✔ |  |  |  | ✔ | ✔ |  | ✔ | ✔ | 6 |
|  | Liu et al.  (2021) [[17](https://doi.org/10.1186/s13046-021-01877-y)] | ✔ |  | ✔ |  |  | ✔ | ✔ |  | ✔ | ✔ | 6 |
|  | Li et al.  (2019) [[25](https://pmc.ncbi.nlm.nih.gov/articles/PMC6945924/)] | ✔ |  |  |  |  | ✔ | ✔ |  | ✔ | ✔ | 5 |
|  | Wang et al.  (2016) [[29](https://doi.org/10.1016/j.gene.2016.03.051)] | ✔ |  | ✔ |  |  | ✔ | ✔ |  | ✔ | ✔ | 6 |
|  | Zhuo et al. (2018) [[30](https://doi.org/10.1016/j.biopha.2018.03.079)] | ✔ |  | ✔ |  |  | ✔ | ✔ |  | ✔ | ✔ | 6 |
|  | Lee et al. (2017) [[38](https://doi.org/10.1016/j.cell.2017.10.037)] | ✔ |  | ✔ |  |  | ✔ | ✔ |  | ✔ |  | 5 |
|  | Moreira et al. (2022) [[45](https://doi.org/10.1016/j.bcp.2022.115161)] | ✔ | ✔ |  |  |  | ✔ | ✔ |  | ✔ | ✔ | 6 |
|  | Moreira et al. (2018) [[46](https://doi.org/10.1016/j.taap.2018.08.012)] | ✔ | ✔ |  |  |  | ✔ | ✔ |  | ✔ | ✔ | 6 |
|  |  |  |  |  |  |  |  |  |  |  |  |  |

**(1)** Publication in peer-reviewed journal, **(2)** statement of control of temperature, **(3)** randomization of treatment or control, **(4)** blinded induction of cancer, **(5)** blinded assessment of outcome, **(6)** Avoidance of anesthetics with significant effects on tumor growth or progression, **(7)** appropriate animal model, **(8)** sample size calculation, **(9)** statement of compliance with regulatory requirements, **(10)** statement regarding possible conflict of interest.
